# Antimicrobial synergy between carprofen and doxycycline against methicillin-resistant *Staphylococcus pseudintermedius* ST71

**DOI:** 10.1186/s12917-016-0751-3

**Published:** 2016-06-24

**Authors:** Rikke Prejh Brochmann, Alexandra Helmfrid, Bimal Jana, Zofia Magnowska, Luca Guardabassi

**Affiliations:** Department of Veterinary Disease Biology, Faculty of Health and Medical Sciences, University of Copenhagen, Stigbøjlen 4, 1870 Frederiksberg C, Denmark; Department of Biomedical Sciences, Ross School of Veterinary Medicine, Basseterre, St Kitts West Indies

**Keywords:** Veterinary antimicrobial therapy, Non-steroidal anti-inflammatory drugs, Multidrug resistance, Dogs

## Abstract

**Background:**

New therapeutic strategies are needed to face the rapid spread of multidrug-resistant staphylococci in veterinary medicine. The objective of this study was to identify synergies between antimicrobial and non-antimicrobial drugs commonly used in companion animals as a possible strategy to restore antimicrobial susceptibility in methicillin-resistant *Staphylococcus pseudintermedius* (MRSP).

**Results:**

A total of 216 antimicrobial/non-antimicrobial drug combinations were screened by disk diffusion using a clinical MRSP sequence type (ST) 71 strain resistant to all six antimicrobials tested (ampicillin, ciprofloxacin, clindamycin, doxycycline, oxacillin and trimethoprim/sulfamethoxazole). The most promising drug combination (doxycycline-carprofen) was further assessed by checkerboard testing extended to four additional MRSP strains belonging to ST71 or ST68, and by growth inhibition experiments.

Seven non-antimicrobial drugs (bromhexine, acepromazine, amitriptyline, clomipramine, carprofen, fluoxetine and ketoconazole) displayed minimum inhibitory concentrations (MICs) ranging between 32 and >4096 mg/L, and enhanced antimicrobial activity of one or more antimicrobials. Secondary screening by checkerboard assay revealed a synergistic antimicrobial effect between carprofen and doxycycline, with the sum of the fractional inhibitory concentration indexes (ΣFICI) ranging between 0.3 and 0.5 depending on drug concentration. Checkerboard testing of multiple MRSP strains revealed a clear association between synergy and carriage of *tetK*, which is a typical feature of MRSP ST71. An increased growth inhibition was observed when MRSP ST71 cells in exponential phase were exposed to 0.5/32 mg/L of doxycycline/carprofen compared to individual drug exposure.

**Conclusions:**

Carprofen restores in vitro susceptibility to doxycycline in *S. pseudintermedius* strains carrying *tetK* such as MRSP ST71. Further research is warranted to elucidate the molecular mechanism behind the identified synergy and its linkage to *tetK*.

## Background

Occurrence of methicillin-resistant staphylococci in animals is a reason for concern in relation to both public and animal health [1]. In small animal veterinary medicine, infections caused by methicillin-resistant *Staphylococcus pseudintermedius* (MRSP) pose a major therapeutic challenge since some MRSP strains, such as the European epidemic clone sequence type (ST) 71, are virtually resistant to all systemic antimicrobial products licensed for use in dogs [2]. As it is unlikely that new antimicrobial classes active against MRSP will enter the veterinary drug market in the near future, new therapeutic strategies are needed to exploit the current antimicrobial arsenal. Combination therapy is one of the possible strategies that can be used to manage severe MRSP infections that cannot be cured by topical antiseptic treatment. Some antimicrobial combinations such as amoxicillin clavulanate and potentiated sulphonamides are widely used in human and veterinary medicine. Research is warranted to identify new combinations of drugs acting on different targets concurrently. It has been hypothesized that combination antimicrobial therapy may prevent or delay development of resistance [3]. Promising results have been shown by combining antimicrobials with small non-antimicrobial helper molecules interfering with resistance [4].

Pharmaceutical preparations targeting eukaryotic cells and used for management of non-infectious diseases, hereafter defined as non-antimicrobial drugs, represent an unexplored source to potentiate existing antimicrobials, restore susceptibility against resistant strains or allow new uses and indications. Various non-antimicrobial drugs have shown in vitro antimicrobial activity [5] but their potential use in combination with existing antimicrobial drugs has never been tested systematically on veterinary pathogens. The objective of this study was to identify synergies between antimicrobial and non-antimicrobial drugs commonly used in small animal veterinary medicine as a possible strategy to restore antimicrobial susceptibility in MRSP. This objective was achieved by i) a double disk diffusion primary screening of six antimicrobial and 36 non-antimicrobial drugs, ii) minimum inhibitory concentration (MIC) testing of selected non-antimicrobials displaying antimicrobial activity and interaction with one or more antimicrobial disk in the primary screening, and iii) checkerboard secondary screening to assess synergy of the selected antimicrobial/non-antimicrobial combinations using a model strain of MRSP ST71 resistant to all antimicrobials tested. The most promising combination was further investigated by growth inhibition analysis and checkerboard testing of additional MRSP strains.

## Methods

### Selection of antimicrobials and non-antimicrobials

Six antimicrobials were selected to represent the five antimicrobial classes most commonly used in dogs and cats: β-lactams [ampicillin (AMP) and oxacillin (OXA)], fluoroquinolones [ciprofloxacin CIP)], lincosamides [clindamycin (CLI)], tetracyclines [doxycycline (DOX)] and potentiated sulfonamides [trimethoprim/sulfamethoxazole (SXT)] [6]. Although amoxicillin is the most frequently used penicillin in clinical practice, AMP was used as a surrogate as recommended by Clinical Laboratory Standard Institute (CLSI) [7]. Similarly, OXA was used for testing methicillin resistance according to CLSI guidelines [7]. Although CIP is not licensed for veterinary use, this fluoroquinolone was used instead of enrofloxacin, which largely metabolized to ciprofloxacin under in vivo conditions [8].

Thirty-six non-antimicrobials used in small animal practice were selected based on data on veterinary usage of drugs in Denmark (VetStat) [9], recommendations on frequency of usage by veterinary professionals at the local university hospital, and availability of the active compounds. Table 1 lists clinical use, solvent and supplier for each non-antimicrobial used in the study.

**Table 1:** List of non-antimicrobial drugs selected for this study

Non-antimicrobial drug	Clinical use	Solvent	Supplier
Prednisolone sodium phosphate	Immunosuppressant	Water	Maymó
Cyclosporine		DMSO	Sigma-Aldrich
Dexamethasone sodium phosphate		Water	Alfasan
Praziquantel	Anthelmintic	DMSO	Haupt Pharma
Ondansetron^a^	Gastrointestinal problems	DMSO	Sigma-Aldrich
Omeprazole		DMSO	Sigma-Aldrich
Ranitidine^a^		Water	Sigma-Aldrich
Metoclopramide^a^		Water	Dechra
Salbutamol/Albuterol	Respiratory problems	Water	Sigma-Aldrich
Fluticasone^a^		DMSO	Sigma-Aldrich
Theophylline^a^		Water	Sigma-Aldrich
Sildenafil^a^		DMSO	Sigma-Aldrich
Bromhexine^a^		DMSO	Zoopan
Carprofen	Pain and inflammation	DMSO	Chanelle
Meloxicam		DMSO	Dopharma
Phenylbutazone^a^		DMSO	Alfasan
Paracetamol/Acetaminophen^a^		Water	SP Veterinaria
Estriol	Urinary problems	DMSO	Haupt Pharma
Medroxy-progesterone actetate	Hormonal problems	DMSO	Alfasan
Levothyroxine		DMSO	Dechra
Thiamazol/Methiamazol		Water	Dechra
Trilostane		DMSO	Dechra
Osaterone		DMSO	Virbac
Methylergometrine/Methylergonovine^a^		Water	Sigma-Aldrich
Levetiracetam^a^	Epilepsy	Water	No data
Pimobendan	Heart failure	DMSO	Dechra
Digoxin^a^		DMSO	Kela
Atenolol^a^		Water	Kela
Captopril^a^		Water	Novartis
Furosemide	Diuretics	DMSO	Alfasan
Spironolactone		DMSO	Haupt Pharma
Clomipramine	Psychological effects	Water	Haupt Pharma
Acepromaxine		Water	Alfasan
Amitriptyline^a^		Water	Haupt Pharma
Fluoxetine hydrochloride^a^		Water	Sigma-Aldrich
Ketoconazole^a^	Antifungals	DMSO	Sigma-Aldrich

^a^Only registered for human use in Denmark

### Bacteria strains and media

MRSP ST71 strain E104 resistant to all six antimicrobials tested was used for primary and secondary screening. Checkerboard testing was extended to four additional MRSP strains including ST71 (E032 and E095) and another widely distributed multidrug-resistant MRSP clone, ST68 (E122 and E135). All strains were grown on blood agar (Oxoid, United Kingdom) and incubated overnight at 37 °C prior to testing. All tests were performed using cation-adjusted Mueller-Hinton agar or broth (Sigma-Aldrich, Germany) using *S. aureus* ATCC 29213 as quality control strain.

### Double disk diffusion test (primary screening)

The strain inoculum was prepared and plated according to CLSI guidelines for disk diffusion [7]. The following disk concentrations were used: AMP (25 μg), CIP (10 μg), CLI (10 μg), DOX (30 μg), OXA (5 μg) and SXT (1.25/25 μg). One antimicrobial disk was tested for each plate. The antimicrobial disk was placed at the centre of the plate and disks impregnated with 20 μL of non-antimicrobial solution at standard concentration (2 g/L) were applied at a 5 mm of distance from the antimicrobial disk. An antimicrobial disk not surrounded by non-antimicrobial disks was used as control on the same plate. Following overnight incubation at 37 °C, the plates were read to detect interactions between antimicrobial and non-antimicrobial disks. A clear extension of the edge of the inhibition zone of the antimicrobial disk in proximity of the non-antimicrobial disk was interpreted as a positive result.

### MIC determination by broth microdilution

The MICs of seven non-antimicrobials displaying interaction with antimicrobial disks in the primary screening were determined by broth microdilution [7]. The following stock concentrations were prepared: bromhexine [8192 mg/L, 75 % dimethyl sulfoxide (DMSO)]; clomipramine, acepromaxine and ketoconazole (1024 mg/L, 20 % DMSO); carprofen (1024 mg/L, 1.6 % DMSO); amitriptyline and fluoxetine (1024 mg/L). Serial two-fold dilutions were prepared in 96-well round-bottom microtiter plates (Thermo Scientific). The range of concentrations tested was determined individually for each compound and ranged between 0.5 and 4096 mg/L.

### Checkerboard assay (secondary screening)

Two-dimensional checkerboard assays [10] were used to assess synergy for seven antimicrobial/non-antimicrobial combinations selected by the primary screening. Carprofen was additionally tested with tetracycline (TET) to check if the synergy effect was antimicrobial class-specific. Fractional Inhibitory Concentration Indexes (FICI) were calculated for each combination to determine whether the effect was truly synergistic (ΣFICI ≤ 0.5), no interaction (ΣFICI >0.5–4) or antagonistic (ΣFICI >4.0) depending on drug concentration [11]. The highest concentration of antimicrobials and non-antimicrobials used for the checkerboard assays was twice the MIC. The highest concentration possible was used if non-antimicrobials could not be dissolved at the desired concentration. Two-fold dilutions were prepared and inoculated with the test strain according to the CLSI guidelines for broth microdilution [7]. After overnight incubation at 37 °C, plates were shaken at 1200 rpm for 1 min in a Bioshake XP (Quantifoil Instruments GmbH). The optical density of growth cultures was measured at 600 nm (OD_600_) using a Powerwave XS (BioTek) operated by software Gen5. Percentages of growth inhibition were calculated for each well using the following equation:

$$ \%\kern0.5em \mathrm{inhibition}=100-\left(\frac{Mean\kern0.5em OD\kern0.5em  of\kern0.5em  treated\kern0.5em  culture}{Mean\kern0.5em OD\kern0.5em  of\kern0.5em  untreated\kern0.5em  culture}\right)\times 100 $$

### Growth inhibition assay

The inhibitory effect of carprofen/DOX was evaluated by exposing the model strain in exponential growth phase to the two drugs alone and in combination. Drug concentrations approximating the peak serum concentration (C_max_) achieved in dogs by standard dosage in single drug therapy were used for this assay. According to the scientific literature, the C_max_ of DOX is 2.74–6.32 mg/L upon oral administration of 5–10 mg/Kg [12], whereas the C_max_ of carprofen is 32.6–38 mg/L upon treatment with 4.0 mg/Kg [13]. Taking into consideration DOX pharmacokinetic (PK) data in dogs and pharmacodynamic (PD) properties against *S. pseudintermedius*, [12] the strain was exposed to 0.5 mg/L DOX and 16, 32 or 64 mg/L carprofen. Briefly, overnight culture of the strain was diluted to 0.05 at an optical density of 600 nm (OD_600_), grown up to OD_600_ 0.4 (10^8^ cell forming units (CFU)/mL) and diluted again 1:1000 (10^5^ CFU/mL). For the next 12 h, samples were collected every hour to perform standard bacterial counts. At OD_600_ 0.1 (10^7^ CFU/mL, approx. after 3.5 h) aliquots of the culture were transferred into small flasks. Individual cultures were exposed to the selected concentrations of each drug alone or in combination and further incubated with untreated control. All cultures were setup in triplicates and incubated in water baths at 37 °C with shaking at 180 rpm. The percentage of growth inhibition at a specific time point was calculated using the following equation:

$$ \%\kern0.5em  inhibition=100-\left(\frac{Mean\kern0.5em CFU\kern0.5em  of\kern0.5em  treated\kern0.5em  culture}{Mean\kern0.5em CFU\kern0.5em  of\kern0.5em  untreated\kern0.5em  culture}\right)\times 100. $$

## Results

Seven of the 36 non-antimicrobial drugs tested in the primary screening were shown to enlarge the edge of the inhibition zone of at least one antimicrobial disk: acepromazine (CLI, OXA), amitriptyline (AMP, OXA), bromexine (OXA), clomipramine (OXA), carprofen (AMP, DOX), fluoxetine (CIP, SXT) and ketoconazole (OXA, SXT). Ketoconazole displayed the highest antibacterial activity (MIC = 32 mg/L), followed by acepromazine, clomipramine and fluoxetine (MIC = 64 mg/L), amitriptyline and carprofen (MIC = 256 mg/L) and bromhexine (MIC > 4096 mg/L).

**Fig. 1:**
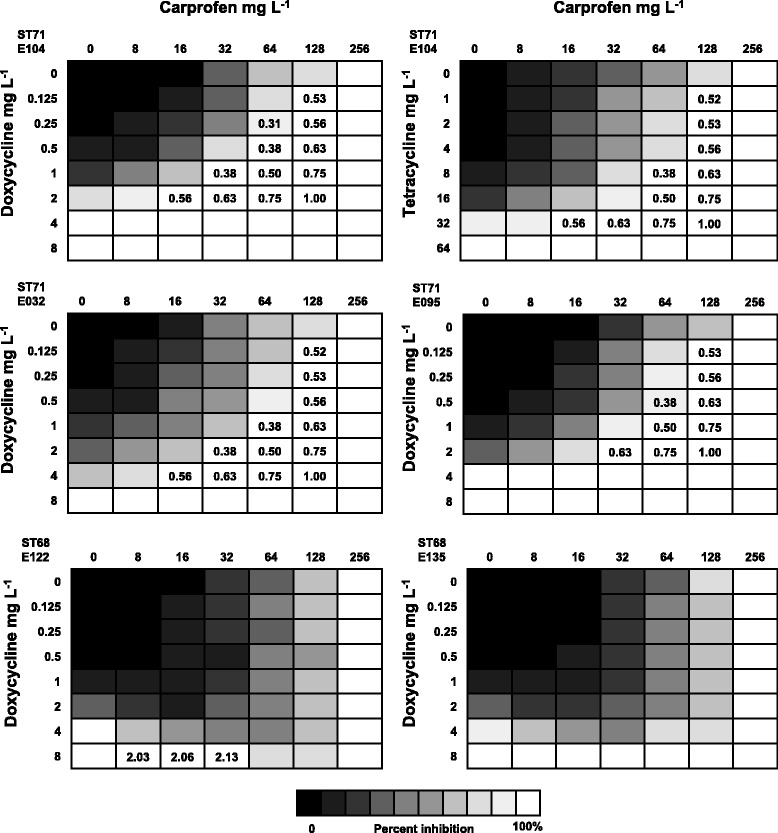
Heat plots of the antibacterial effects of DOX or TET in combination with carprofen on three methicillin-resistant *Staphylococcus pseudintermedius* ST71 strains (E104, E032 and E095) and two ST68 strains (E122 and E135). The level of growth inhibition is expressed by colour intensity: the more intense the colour is, the less inhibited the strain was. The Fractional Inhibitory Concentration Index (FICI) was calculated for wells with no visible growth (white colour). Indexes of ΣFICI ≤ 0.5 correspond to synergistic effect, ΣFICI >0.5–4 no effect, ΣFICI > 4 antagonistic. The heat plots show the average of three biological replicates

These seven non-antimicrobials were further tested by checkerboard assays in combination with the antimicrobial displaying the largest inhibition zone in the double disk diffusion assay. The ΣFICI of bromhexine was not determined because the drug could not be dissolved at a sufficient concentration to determine the MIC (MIC > 4096 mg/L). Carprofen displayed synergistic antimicrobial activity with DOX, whereas the other five antimicrobial/non-antimicrobial combinations showed no interaction (ΣFICI = 1.01–1.35). Additional checkerboard assays were performed to determine at which drug concentrations carprofen displayed synergy with DOX or TET. Synergy was observed in presence of 64 mg/L of carprofen and 0.25–1 mg/L DOX (ΣFICI = 0.31–0.5). At lower carprofen concentration (32 mg/L), synergy was only displayed in presence of 1 mg/L of DOX (ΣFICI index = 0.38), whereas at higher concentration (128 mg/L) no effect was observed in presence of 0.125–1 mg/L of DOX (ΣFICI = 0.53–0.75). No effect was observed by increasing the concentration of DOX up to 2 mg/L with carprofen concentrations ranging from 16 to 64 mg/L (ΣFICI = 0.56–0.75). The synergy patterns of carprofen and TET were similar to those observed for DOX, even though they were less pronounced and required higher antibiotic concentrations (8–16 mg/L), resulting in a ΣFICI between 0.38 and 0.5 (Fig. 1). Checkerboard testing of four additional MRSP strains (two ST71 and two ST68) revealed synergy for the two ST71 strains when DOX and carprofen were combined at concentrations of 0.5/64, 1/64, 2/64 or 2/32 mg/L. On the contrary, no synergy was observed for the two ST68 strains (Fig. 1). At DOX/carprofen concentrations achievable in dogs by single drug therapy (0.5/32 mg/L), the percentage of growth inhibition measured by spectrophotometry was 54 % higher for the three ST71 than for the two ST68 strains.

**Fig. 2:**
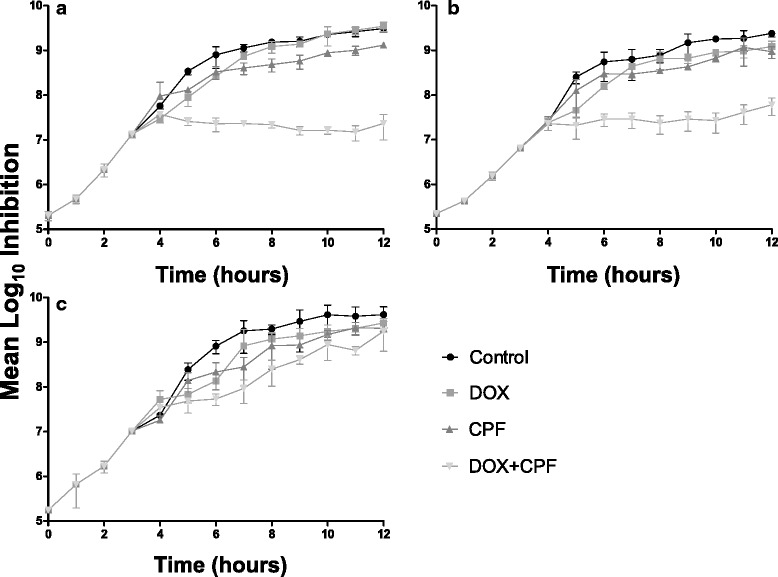
Growth inhibition of methicillin-resistant *Staphylococcus pseudintermedius* E104 exposed to doxycycline (0.5 mg/L DOX, filled squares), carprofen (CPF, filled triangle pointing upwards) and the combination of the two drugs (DOX + CPF, filled triangle pointing downwards) using three concentrations of CPF (**a**: 64 mg/L; **b**: 32 mg/L; **c**: 16 mg/L). The control (filled circles) was treated with neither DOX nor CPF. Error bars denote standard deviation (SD) of three biological replicates

Growth inhibition experiments were performed to assess the effect of carprofen and DOX, individually and in combination, during exponential growth of the model strain MRSP E104. Based on viable cell counts, the effect of DOX and carprofen alone were significantly lower than for the combination of the two drugs (Fig. 2). After 4.5 h of exposure to 0.5 mg/L of DOX, the growth of strain was inhibited by 8.3 %; exposure to 16, 32 or 64 mg/L of carprofen alone resulted in 6.2, 26.6 and 40.9 % growth inhibition, respectively; exposure to 0.5 mg/L of DOX in combination with 16, 32 or 64 mg/L of carprofen inhibited growth by 45.7, 89.5 and 100 %, respectively, indicating a clear synergistic effect on growth inhibition by the drug combination.

## Discussion

This study indicates that approximately 19 % of the 36 non-antimicrobial drugs tested were able to potentiate the antibacterial activity of one or more known antimicrobials against MRSP. All the seven non-antimicrobials that were found to have antimicrobial-potentiating activity (acepromazine, amitriptyline, bromexine, carprofen, clomipramine, fluoxetine and ketoconazole) have been previously reported to possess antibacterial activity [14–20]. Carprofen was shown to be particularly interesting to potentiate the antimicrobial activity of DOX as it displayed synergy at drug concentrations that may be achieved during therapy in dogs. Carprofen is a non-steroidal anti-inflammatory drug (NSAID) for veterinary treatment of inflammation and pain management. It has earlier been reported to have a clinical effect when used in combination with tilmicosin for antimicrobial therapy of bovine respiratory disease [21]. DOX is the most widely used tetracycline in small animal practice due to low systemic toxicity [22] and higher antimicrobial activity compared to TET [12]. However, due to widespread tetracycline resistance, DOX is presently regarded as a second choice antibiotic for most indications except upper respiratory tract infections [23].

The synergy between carprofen and DOX was studied in multiple MRSP strains, leading to the identification of an association between the synergistic effect of this drug combination and strains belonging to the clonal lineage ST71, which harbours the efflux pump-mediated tetracycline resistance gene *tetK* [2]. In contrast, no synergy was observed for MRSP strains belonging to ST68, which are consistently associated with *tetM,* [2] an unrelated tetracycline resistance gene encoding ribosomal protection of the drug target. The association between *tetK* and DOX/carprofen synergy was further illustrated by the analysis of strain cultures exposed to concentrations of DOX/carprofen achievable in dogs by single therapy (0.5/32 mg/L), which showed a significantly higher growth inhibition in the three strains harbouring *tetK* compared to the two strains containing *tetM*. These results suggest that DOX/carprofen synergy only occurs in strains carrying *tetK*. The molecular mechanism behind the identified synergy and its linkage to *tetK* remains unknown. Such synergy mechanism is unlikely linked to the bactericidal effect of carprofen on DNA replication, suggesting that carprofen interacts with multiple targets in the bacterial cell. Various mechanisms are possible, including inhibition of *tetK* gene expression, blockage of the TetK efflux pump or interference with the energy source used by TetK to pump DOX out of the cell.

The recommended dosage for oral administration of carprofen in dogs is 4 mg/Kg of body weight daily. This dosage leads to peak plasma concentration of 35.30 ± 2.70 mg/L at 1.25 ± 0.25 h [13]. Higher dosages up to 9 mg/Kg were shown to be well tolerated in healthy beagles [13]. These data suggest that the carprofen (32 mg/L) concentration required for synergy with DOX (0.5 mg/L) can be achieved in vivo. However, there may be marked differences between in vitro and in vivo conditions due to serum protein binding, which affects drug’s efficiency. Further PD/PK studies are needed to assess the therapeutic potential of DOX/carprofen, including in vitro experiments assessing the effects of canine serum protein binding on carprofen activity. In an earlier study by Brentnall et al. [24] the influence of oxytetracycline on carprofen PD and PK was evaluated for therapy of bacterial pneumonia in calves, indicating that no alteration to carprofen dosage is required when the two drugs are co-administered. There is an obvious rationale for investigating the use of NSAIDs in combination to DOX for some canine infections, such as upper respiratory tract infections. Carprofen analogues able to establish synergy with DOX at lower concentrations could be developed to facilitate translation of the results of this study into veterinary clinical practice. Furthermore, since MRSP is a common cause of skin and soft tissue infections, carprofen/DOX formulations could be developed for topical use, which may allow achievement of higher carprofen concentrations at the infection site. Interestingly, DOX has earlier been reported to have anti-inflammatory effects [25]. Thus, the combination of the two drugs might also have enhanced anti-inflammatory activity compared to single therapy.

The interactions between non-antimicrobial drugs and tetracyclines have occasionally been explored for potential clinical applications in human medicine. One example is the recent study by Ejim et al. [26] describing the synergistic effect of minocycline in combination with loperamide, a medication used for control of diarrhoea. Our study is the first attempt to investigate this alternative avenue for possible veterinary clinical applications.

## Conclusion

The results show that carprofen is a potential antimicrobial helper drug to restore susceptibility to DOX in DOX-resistant MRSP strains carrying *tetK*. This finding is of clinical relevance since the epidemic multidrug-resistant clone MRSP ST71 is virtually resistant to all antimicrobial drugs licensed for veterinary use and has been previously shown to carry consistently *tetK* as the only tetracycline resistance determinant. More research is needed in order to understand the mode of action of this drug combination as well as to assess the clinical potential of carprofen as a DOX helper drug in small animal medicine.

## Abbreviations

AMP, ampicillin; CIP, ciprofloxacin; CLI, clindamycin; CLSI, Clinical Laboratory Standards Institute; DMSO, dimethyl sulfoxide; DOX, doxycycline; FICI, fractional inhibitory concentration index; MIC, minimum inhibitory concentration; MRSP, methicillin-resistant *Staphylococcus pseudintermedius*; OXA, oxacillin; ST, sequence type; SXT, trimethoprim/sulfamethoxazole; TET, tetracycline
